# The efficacy of mesalazine on nonspecific terminal ileal ulcers: A randomized controlled trial

**DOI:** 10.3389/fphar.2022.989654

**Published:** 2022-09-23

**Authors:** Junrong Li, Fangmei Ling, Di Guo, Jinfang Zhao, Ling Cheng, Yidong Chen, Mingyang Xu, Liangru Zhu

**Affiliations:** ^1^ Division of Gastroenterology, Union Hospital, Tongji Medical College, Huazhong University of Science and Technology, Wuhan, China; ^2^ Department of Geriatrics, Wuhan Central Hospital, Wuhan, China; ^3^ Center for Life Sciences, Tsinghua University, Beijing, China; ^4^ Department of Gastroenterology, The First Peoples Hospital of Nanyang City, Henan, China

**Keywords:** randomized controlled trial, nonspecific ulcers, terminal ileum, mesalazine, efficacy

## Abstract

**Background:** Nonspecific terminal ileal ulcers are one of the common ulcerative diseases in terminal ileum. However, the studies about treatment efficacy are scarce. We aimed to investigate the efficacy of mesalazine in the treatment of this disease.

**Methods:** Eighty-two patients with nonspecific terminal ileal ulcers who sought outpatient medical treatment in the Division of Gastroenterology, Wuhan Union Hospital, from April 2016 to January 2019 were enrolled and randomly divided into two groups. The experimental group took mesalazine orally, 4.0 g/d, once a day for 3 months. The control group was followed up without special intervention. The primary endpoint was the endoscopic remission rate at the 6^th^ and 12^th^ month. Secondary endpoints included the clinical remission rate at the 1^st^, 6^th^ and 12^th^ month and adverse events (ChiCTR1900027503).

**Results:** About the endoscopic efficacy, the remission rate of the experimental group and control group was 73.2 versus 61.0% at the 6^th^ month (RR = 1.20, 95%CI 0.88∼1.63, *p* = 0.24) and 87.8 versus 78.0% at the 12^th^ month (RR = 1.13, 95%CI 0.92∼1.37, *p* = 0.24). About the clinical efficacy, the remission rate was 70.3 versus 43.8% at the 1^st^ month (RR = 1.61, 95%CI 1.03∼2.51, *p* = 0.03), 83.8 versus 68.8% at the 6^th^ month (RR = 1.22, 95%CI 0.93∼1.60, *p* = 0.14) and 91.9 versus 81.3% at the 12^th^ month (RR = 1.13, 95%CI 0.93∼1.37, *p* = 0.34). During follow-up, no patients were diagnosed with Crohn’s disease or intestinal tuberculosis, and no patients developed significant complications.

**Conclusion:** For patients with nonspecific terminal ileal ulcers, there is no disease progression over a short term. In addition, there is no significant difference in clinical or endoscopic efficacy between patients who received mesalazine and patients who are followed up without special intervention.

## 1 Introduction

In recent years, due to the deepening awareness in intestinal diseases, the wide application of endoscopy, capsule endoscopy and other endoscopic techniques, and the improvement in endoscopic surgical and diagnostic skills, the detection rate and diagnosis rate of terminal ileal ulcers have been significantly improved (Jeong et al., 2008; Greaves and Pochapin, 2006; Courville et al., 2009). The terminal ileum is a common site for small intestinal lesions. Crohn’s disease (CD), intestinal tuberculosis (ITB), nonsteroid anti-inflammatory drug (NSAID)-related enteropathy and nonspecific terminal ileal ulcers are common ulcerative diseases occurring at the end of ileum (Goulart et al., 2016). Nonspecific terminal ileal ulcers are a chronic ulcerative disease located in the terminal ileum. The understanding of this disease is still incomplete, such as the pathogenesis, clinical characteristics, diagnosis and treatment, and they need to be further studied. A study showed that there were no specific gastrointestinal symptoms or signs in patients with nonspecific terminal ileal ulcers, which were thus easily ignored by clinicians. Some patients even had no obvious clinical manifestations, and ulcers were found during routine endoscopic examinations (Kim et al., 2021). Currently, there is no standard treatment for patients with nonspecific terminal ileal ulcers (Karnam et al., 2001).

Mesalazine, also known chemically as 5-aminosalicylic acid (5-ASA), is commonly used in the treatment of ulcerative colitis (UC). Mesalazine exerts an anti-inflammatory effect on the intestinal wall after taken orally. Because of the anti-ulcer and antioxidant efficacy, mesalazine is not only used in the treatment of UC, but also in other diseases (Beiranvand, 2021). A research showed that mesalazine significantly attenuated NSAID-induced mucosal injury in patients with small bowel enteropathy (Rácz et al., 2013). In addition, a recent study showed that 5-ASA also exerted ameliorative and protective effects on ethanolic gastric ulcers in experimental rats by strengthening the antioxidant defense system of gastric mucosal cells (Beiranvand and Bahramikia, 2020). Relevant studies revealed that mesalazine yielded mild adverse reactions and was a relatively safe drug (Klotz, 2012). Therefore, mesalazine might be an effective and safe drug for nonspecific terminal ileal ulcers. At present, the relevant studies are scarce. Mesalazine was used in the treatment of patients with nonspecific small intestinal ulcers in a study and showed that the patient symptoms improved to varying degrees. However, the disappearance of ulcers in patients was not associated with the use of mesalazine (Wang et al., 2011). Based on the above background, this study focused on the clinical and endoscopic efficacy of mesalazine in treating patients with nonspecific terminal ileal ulcers and analyzed the clinical, endoscopic and histopathological characteristics of the disease. The primary endpoint was the endoscopic remission rate at the 6^th^ and 12^th^ month. The secondary endpoints were clinical remission rate at the 1^st^, 6^th^ and 12^th^ month and adverse events.

## 2 Materials and methods

### 2.1 Study design

This was an observer-blinded, prospective randomized-controlled trial. The study was approved by the Ethics Committee of Tongji Medical College, Huazhong University of Science and Technology, Ethics No. 2018-S (493), and was registered on the Chinese Clinical Trial Registry website with the registration number of ChiCTR1900027503. The sample size was calculated according to the remission rate from the previous studies and experience before our study. The remission rate in the experimental group and control group was anticipated to be 90 and 65%, respectively. The estimated sample size was 32 patients per group with a risk of 0.05 and a power of 0.80, using PASS 15.0 software. Considering 20% dropout, at least 40 subjects in each group were needed in this study.

### 2.2 Study subjects

A total of 86 patients with nonspecific terminal ileal ulcers sought outpatient medical treatment in the Division of Gastroenterology, Union Hospital, Tongji Medical College, Huazhong University of Science and Technology from April 2016 to January 2019, among whom 4 patients under 18 years old were excluded. The remaining 82 patients were randomly assigned into either the experimental group or the control group by computer-generated randomization. In the experimental group, 41 patients were given mesalazine tablets orally at 4.0 g/d, and once a day for 3 months (drug manufacturer: FERRING INTERNATIONAL CENTER SA, Import Drug Registration No. H20181183). Forty-one cases in the control group were observed and followed up without special intervention, and a light diet was highlighted in both groups.

### 2.3 Inclusion and exclusion criteria

Inclusion criteria: ① Patients signed their informed consent voluntarily; ② >18 years old, no limitation on sex; ③ Endoscopy revealed ulcers in the terminal ileum or terminal ileitis, with no limitations on size or number of ulcers; ④ No ulcers were found in other parts of the intestinal tract by computed tomography enterography (CTE), magnetic resonance enterography (MRE) or capsule endoscopy within 1 month. ⑤ Pulmonary computed tomography (CT) imaging, purified protein derivative (PPD), T-cell spot test (T-SPOT) and tuberculosis antibody results were normal; ⑥ The patients tested negative for cytomegalovirus DNA and Epstein-Barr virus DNA; ⑦ Histopathology showed nonspecific ulcers, and the surrounding mucosa showed nonspecific inflammatory changes, with no granulomas or crypts change.

Exclusion criteria: ① Patients with ulcerative disease in terminal ileum such as CD, ITB, ischemic enteropathy, infectious (bacterial, viral, fungal) enteritis, and eosinophilic enteritis; ② Patients with characteristics of CD such as fistulas, perianal lesions, skip lesions under endoscopy or imaging, longitudinal/cobblestone appearance ulcers, transmural inflammation or non-caseating granuloma. ③ Patients with caseating granuloma or positive stain/culture for acid fast-bacillus. ④ Patients who received NSAIDs, potassium chloride tablets, diuretics or herbal remedy in the last 6 months; ⑤ Patients with previous abdominal surgery; ⑥ Patients with digestive system tumors; ⑦ Patients who were pregnant or lactating; ⑧ Patients who were allergic to salicylic acid drugs.

### 2.4 Follow-up and outcomes

The primary endpoint was the endoscopic remission rate at the 6^th^ and 12^th^ month. Secondary endpoints included the clinical remission rate at the 1^st^, 6^th^ and 12^th^ month and adverse events. All patients were followed up for 12 months. If symptoms disappeared completely and no related complications occurred, then the clinical efficacy was determined to be cured. If symptoms improved without affecting the patients’ daily life or work, then the clinical efficacy was determined to be improved. If there was no improvement or symptoms were aggravated, then the clinical efficacy was determined to be ineffective. Endoscopy images were read by two experienced endoscopists who did not know about the grouping of patients. If there were no erosions, ulcers, congestion or edema in the terminal ileum mucosa, then it was determined to be cured. If the lesions were fewer or smaller, they were considered to be improved. If the endoscopic appearance was not improved or even worse, the treatment was deemed ineffective. Remission rate equaled cured rate plus improved rate.

### 2.5 Statistical analysis

Statistical analysis was performed using SPSS 23.0. For numerical data, those conforming to a normal distribution were expressed as the mean ± standard deviation. Those conforming to a skewed distribution were represented by the median. A t test was used for data conforming to a normal distribution, and a nonparametric test was used for comparisons between groups for data conforming to a skewed distribution. Categorical variables were presented as the number and percentage of patients and were analyzed by the chi-square test or Fisher’s exact test. Results were expressed as risk ratios (RR) with 95% confidence intervals (CI). *P* < 0.05 was considered statistically significant.

## 3 Results

Eighty-two patients were finally enrolled, including 52 males (63.4%) and 30 females (36.6%), with an average age of 42.84 ± 12.73 years (range, 18∼70) and a median course of 24 months (range, 1∼312). The baseline characteristics were compared in Table 1.

**TABLE 1:** Baseline features of the patients, compared between 2 groups.

Patients		Experimental group	Control group	*p*
Male-female ratio		1.93:1	1.56:1	0.65
Average age (years)		43.71 ± 13.44	41.98 ± 12.08	0.54
Median course (months)		12	24	0.06
	current	10	7	
Smoking	past	5	6	0.71
	never	26	28	
	current	12	11	
Drinking	past	2	0	0.47
	never	27	30	
	abdominal pain	26	21	
Clinical manifestations	diarrhea	6	9	0.65
	abdominal distension	9	6	
	shapeless stools	6	7	
	constipation	2	5	
	bloody stools	4	2	
	tenesmus	1	2	
	mucous stools	1	2	
	no symptoms	4	9	

**FIGURE 1:**
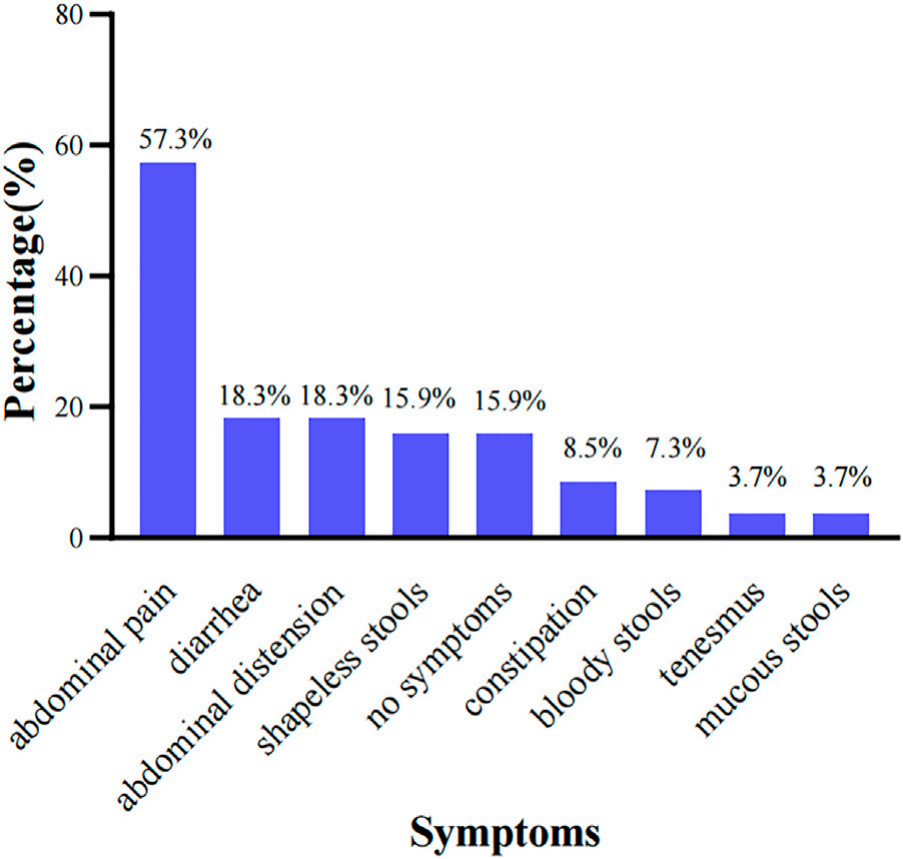
Clinical manifestations in patients with nonspecific terminal ileal ulcers (*n* = 82).

The clinical manifestations included abdominal pain in 47 cases (57.3%), diarrhea in 15 cases (18.3%), abdominal distension in 15 cases (18.3%), shapeless stools in 13 cases (15.9%), constipation in 7 cases (8.5%), bloody stools in 6 cases (7.3%), tenesmus in 3 cases (3.7%) and mucous stools in 3 cases (3.7%). Thirteen patients (15.9%) had no obvious clinical manifestations. Clinical manifestations were shown in Figure 1.

**FIGURE 2:**
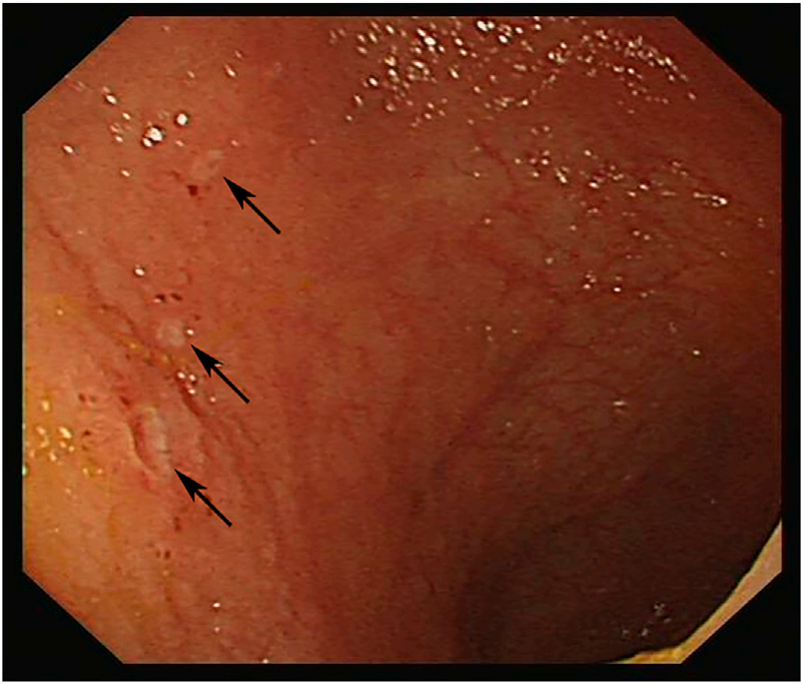
Endoscopy image: Multiple superficial ulcers with white moss in the terminal ileum.

**FIGURE 3:**
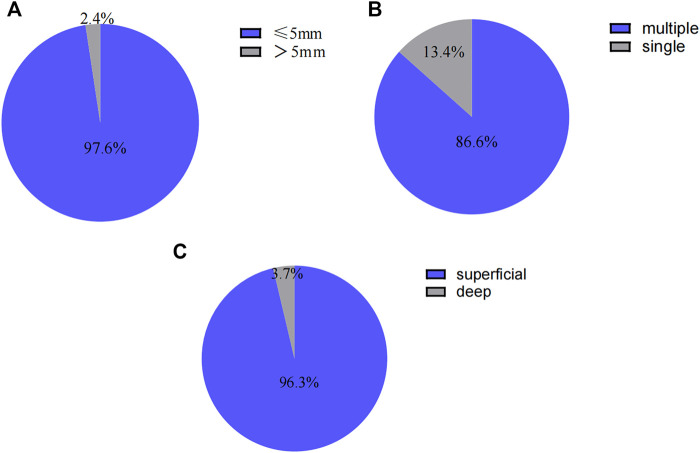
**(A)** Endoscopic features in patients with nonspecific terminal ileal ulcers (ulcer size). **(B)** Endoscopic features in patients with nonspecific terminal ileal ulcers (ulcer number). **(C)** Endoscopic features in patients with nonspecific terminal ileal ulcers (ulcer depth).

Endoscopic manifestations included ulcers or erosions, with hyperemia and edema in the terminal ileum mucosa (one representative case was shown in Figure 2). In addition, the size of ulcers were ≤ 5 mm in 80 patients (97.6%), multiple in 71 cases (86.6%) and superficial in 79 cases (96.3%). The endoscopic features including ulcer size, number and depth were shown in Figures 3A–C, respectively.

**FIGURE 4:**
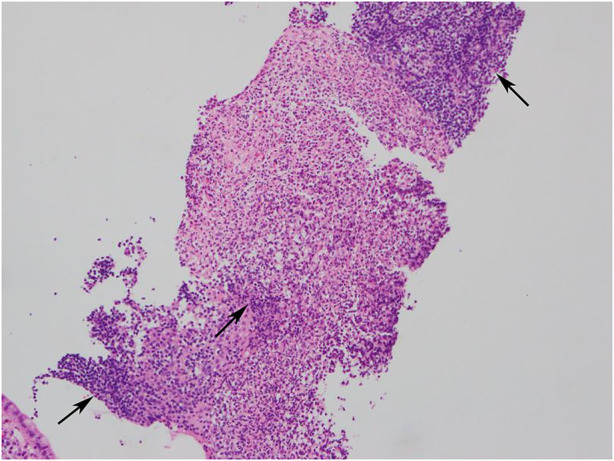
Histopathology image (HEx100): Chronic inflammatory changes in mucosal tissues, lymphocytic infiltration and numerous inflammatory cells exudation and necrosis.

In this study, the histopathological manifestations revealed chronic inflammatory changes in the terminal ileum mucosal tissues, which may be accompanied by lymphoproliferative tissues. and there were no granulomatous lesions or crypts change (one representative case was shown in Figure 4).

### 3.1 Primary outcome

At the 6^th^ month, 8 cases were cured, 22 cases were improved, 11 cases were ineffective, and the endoscopic remission rate was 73.2% in the experimental group. In the control group, 7 cases were cured, 18 cases were improved, 16 cases were ineffective, and the endoscopic remission rate was 61.0%. There was no statistically significant difference in endoscopic efficacy (RR = 1.20, 95%CI 0.88∼1.63, *p* = 0.24). At the 12^th^ month, 9 cases were cured, 27 were improved, 5 were ineffective, and the endoscopic remission rate was 87.8% in the experimental group. In the control group, 10 cases were cured, 22 were improved, 9 were ineffective, and the endoscopic remission rate was 78.0%. There was no statistically significant difference in endoscopic efficacy (RR = 1.13, 95%CI 0.92∼1.37, *p* = 0.24). The comparison of endoscopic efficacy between two groups was shown in Table 2.

**TABLE 2:** Endoscopic efficacy comparison of the patients between 2 groups (*n* = 82).

Follow-up	Groups	Remission rate (%)		Ineffective rate (%)	*p*	RR (95%CI)
		Cured rate (%)	Improved rate (%)			
6^th^ month	Experimental group	8 (19.5)	22 (53.7)	11 (26.8)	0.24	1.20 (0.88∼1.63)
	Control group	7 (17.1)	18 (43.9)	16 (39.0)		
12^th^ month	Experimental group	9 (22.0)	27 (65.9)	5 (12.2)	0.24	1.13 (0.92∼1.37)
	Control group	10 (24.4)	22 (53.7)	9 (22.0)		

### 3.2 Secondary outcomes

#### 3.2.1 Clinical remission rate

There were no obvious clinical manifestations in 13 patients (4 in the experimental group and 9 in the control group), so they were not included in the clinical efficacy evaluation. In the experimental group, 7 cases were cured, 19 cases were improved, 11 cases were ineffective, and the clinical remission rate was 70.3% at the 1^st^ month. In the control group, 5 cases were cured, 9 cases were improved, 18 cases were ineffective, and the clinical remission rate was 43.8% at the 1^st^ month. The difference in clinical efficacy between the two groups was statistically significant (RR = 1.61, 95%CI 1.03∼2.51, *p* = 0.03). At the 6^th^ month in the experimental group, 9 patients were cured, 22 were improved, and 6 had ineffective treatment, and the clinical remission rate was 83.8%. In the control group, 6 cases were cured, 16 cases were improved, 10 cases were ineffective, and the clinical remission rate was 68.8%. There was no statistically significant difference in clinical efficacy (RR = 1.22, 95%CI 0.93∼1.60, *p* = 0.14). At the 12^th^ month, 11 patients in the experimental group were cured, 23 were improved, 3 had ineffective treatment, and the clinical remission rate was 91.9%. In the control group, 8 cases were cured, 18 cases were improved, 6 cases were ineffective, and the clinical remission rate was 81.3%. There was no statistically significant difference in clinical efficacy between the two groups (RR = 1.13, 95%CI 0.93∼1.37, *p* = 0.34) as shown in Table 3.

**TABLE 3:** Clinical efficacy comparison of the patients between 2 groups (*n* = 69).

Follow-up	Groups	Remission rate (%)		Ineffective rate (%)	*p*	RR (95%CI)
		Cured rate (%)	Improved rate (%)			
1^st^ month	Experimental group	7 (18.9)	19 (51.4)	11 (29.7)	0.03	1.61 (1.03∼2.51)
	Control group	5 (15.6)	9 (28.1)	18 (56.3)		
6^th^ month	Experimental group	9 (24.3)	22 (59.5)	6 (16.2)	0.14	1.22 (0.93∼1.60)
	Control group	6 (18.8)	16 (50.0)	10 (31.3)		
12^th^ month	Experimental group	11 (29.7)	23 (62.2)	3 (8.1)	0.34	1.13 (0.93∼1.37)
	Control group	8 (25.0)	18 (56.3)	6 (18.8)		

#### 3.2.2 Adverse events

In the experimental group, only 2 patients showed slight abdominal distension and nausea, respectively, which was not serious enough to stop the medication.

## 4 Discussion

Nowadays, terminal ileal ulcers are increasingly common under endoscopy. A study included 1497 patients who underwent ileoceroscopy, and found that 74 patients (5.0%) had terminal ileal ulcers (Mehta et al., 2017). Terminal ileal ulcers may be caused by a wide variety of diseases, including CD, NSAID, ITB, eosinophilic enteritis and so on (Dilauro and Crum-Cianflone, 2010). In addition, there are a significant part of patients with nonspecific terminal ileal ulcers. For example, in the previously mentioned study, about 40% of 74 patients were diagnosed with this disease (Mehta et al., 2017). Nonspecific terminal ileal ulcers are a nonspecific ulcer of the small intestine that occurs in the terminal ileum and does not involve the rest part of the small intestine or the upper digestive tract, which pathogenesis is still unclear. Due to the long disease course, some patients were misdiagnosed as functional bowel disease before being diagnosed with nonspecific terminal ileal ulcers (Wang et al., 2011).

A study showed that the common clinical manifestations of patients with nonspecific terminal ileal ulcers included abdominal pain, diarrhea, abdominal distension, constipation and bloody stools. Besides, fever and weight loss were less common than patients with CD and ITB (Kedia et al., 2016). In this study, most patients presented with abdominal pain, diarrhea, abdominal distension, shapeless stools, constipation or bloody stools, and no patients presented with fever or significant weight loss, which was consistent with the previous study (Zhong et al., 2020). The endoscopic manifestations included multiple, superficial and small ulcers without intestinal stricture or malformation. Histopathological manifestations revealed nonspecific inflammation, without granulomas, eosinophil infiltration or viral inclusions. In addition, the endoscopic manifestations were not always parallel to the clinical manifestations, and there were no new clinical manifestations or complications such as intestinal perforation or intestinal obstruction during a follow-up period of 7 years (Wang et al., 2011). Therefore, it was suggested in some studies that routine biopsy was not required for patients with terminal ileal ulcers who did not consider the diagnosis of IBD (Velidedeoğlu et al., 2015). In this study, 82 patients presented with nonspecific endoscopic and histopathological manifestations, with a benign disease course, which was basically consistent with other related studies.

No effective medications are validated for the treatment of nonspecific terminal ileal ulcers, and symptomatic treatment is the main choice in clinical. A study indicated that the symptoms and endoscopic manifestations in some patients could be improved to varying degrees when they were observed and followed up without medications (Kim et al., 2021). Mesalazine has been used in the treatment of UC since the 1940s, and is currently a commonly used medication for mild to moderate UC (Chibba and Moss, 2020). It was reported that the mechanisms of action may include blocking the production of proinflammatory factors, downregulating the production of anti-angiogenic factors and promoting the healing of intestinal epithelial wounds (MacDermott, 2000; Desreumaux and Ghosh, 2006; Lyakhovich and Gasche, 2010). The adverse reactions of mesalazine are minor and generally well tolerated in patients of different age groups, without dose-related side effects (Sehgal et al., 2018; Cuffari et al., 2016). Therefore, mesalazine may be an effective medication for the treatment of nonspecific terminal ileal ulcers. However, the related studies are scarce, and especially randomized controlled trials about mesalazine in the treatment of nonspecific terminal ileal ulcers are currently lacking. There was a study in which 2 patients with nonspecific small intestinal ulcers were treated orally with symptomatic treatment including mesalazine, and their symptoms improved to varying degrees, but the ulcers persisted or recurred (Wang et al., 2011). In our study, among 41 patients treated with mesalazine, 30 cases (73.2%) and 36 cases (87.8%) achieved endoscopic remission at the 6^th^ and 12^th^ month, respectively, which was not significantly different from 25 cases (61.0%) and 32 cases (78.0%) in the control group. Similarly, there was no significant difference in clinical remission rate at the 6^th^ and 12^th^ month between two groups. Therefore, regular follow-up without medications might be a better choice for patients with nonspecific terminal ileal ulcers in clinical practice.

There were several advantages in our study. Firstly, this is the first research to focus on the clinical and endoscopic efficacy of mesalazine in treating patients with nonspecific terminal ileal ulcers. And our study was a prospective randomized controlled trial, with accurate data and small bias. In addition, repeated clinical and endoscopic follow-up within 12 months demonstrated the prognosis of patients in different periods, as well as the efficacy of mesalazine compared with the control. However, our study also has the following limitations: insufficient patient enrollment and follow-up period; the endoscopic remission rate at the 1^st^ month could not be analyzed because most patients were reluctant to undergo the preparation process for colonoscopy. Besides, patients in the control group received no drugs, and it is obvious for them to know the grouping, so psychological factors cannot be ruled out for the results. Multi-center, large-scale and long-term prospective randomized controlled trials are needed for further study.

## 5 Conclusion

Common clinical manifestations in patients with nonspecific terminal ileal ulcers include abdominal pain, diarrhea, abdominal distension, constipation and bloody stools, and about 16% of patients have no obvious clinical manifestations. Endoscopic manifestations include ulcers or erosions, with hyperemia and edema in the terminal ileum mucosa. Histopathology shows chronic inflammatory changes in the terminal ileum mucosal tissues. No new symptoms or intestinal complications occurs during the 12-month follow-up. In addition, there is no significant difference in clinical or endoscopic efficacy between patients who receive mesalazine and patients who are followed up without special intervention, which needs to be further explored in future clinical studies.

## Data Availability

The original contributions presented in the study are included in the article/supplementary material, further inquiries can be directed to the corresponding author.
